# Life cycle inventories and environmental impact datasets for the semi-industrial processing of food Faba bean or Pea protein ingredients

**DOI:** 10.1016/j.dib.2025.112247

**Published:** 2025-11-07

**Authors:** Fanny Guyomarc’h, Simone Scussat, Jean-Eudes Hermant, Claire Berton-Carabin, Marc Anton, Karine Gallardo-Guerrero, Rémi Saurel

**Affiliations:** a INRAE-Institut Agro, UMR 1253 Science et Technologie du Lait et de l’Œuf (STLO), Rennes, France; b IMPROVE SAS, rue du Fond Lagache, Dury, France; c INRAE, UR 1268 Biopolymères Interactions Assemblages (BIA), Nantes, France; d Université de Bourgogne Europe, Institut Agro Dijon, INRAE, UMR 1347 Agroécologie, Dijon, France; e Institut Agro, INRAE, Université de Bourgogne, UMR 02102 Procédés Alimentaires et Microbiologiques (PAM), Dijon, France

**Keywords:** Life cycle assessment, Life cycle inventory, Plant-based protein ingredients, Faba bean, Pea

## Abstract

Thanks to their agronomical, nutritional and technological interests, pulses are very promising to reach the food-related sustainable development goals (SDG). In developed countries, they typically appear as good substitutes to animal proteins, thereby relieving livestock’s environmental impacts of food systems. However, their share in the Western diet has dramatically declined throughout the 20th century. As a consequence, the release of public data regarding processed pulses is slower than for animal-based food products, while comprehensive comparative evaluation capabilities for animal and plant proteins are deemed necessary. Tremendous effort is therefore currently invested to support the reintroduction of pulses in agricultural practices and food innovations. In this way, life cycle assessment (LCA) is a standard method that quantifies the potential environmental impacts of a manufactured product. In compliance with ISO 14 040 principles and framework, this data paper provides life cycle inventory and life cycle assessment datasets related to the production of two different types of pulse protein ingredients: dry-fractionated protein concentrates from five faba bean varieties and one pea variety, and a wet-fractionated protein isolate from one of the five faba bean varieties. Processing was achieved at a semi-industrial scale using classical technologies used in France in the 2020s. Background data was chosen in the Agribalyse 3.1.1 and Ecoinvent 3.9 databases. In the presented semi-industrial protein concentrate production system, the seeds were sorted, cleaned, dehulled then ground and turbo separated into a protein-rich and a starch-rich fractions. This data paper therefore provides the allocation factors to apply mass, dry matter, protein or economic allocation at dehulling and turbo separation. In the presented semi-industrial protein isolate production system, globulin proteins were extracted from an intermediate flour product by dispersion in water, acid precipitation, neutralization and stabilization into powder. All side streams were regarded as wastes and therefore no allocation was applied. Finally, the data paper provides the potential environmental impacts of 1 kg of each product using the characterization method EF 3.0 and the different allocation rules.

Specifications Table

**Table:** 

Subject	Earth & Environmental Sciences
Specific subject area	Environmental assessment of food processes for the manufacture of pulse-based ingredients
Type of data	Table, Figure Raw, Analyzed.
Data collection	Foreground data were collected at the IMPROVE SAS pilot plant during the winter 2023–2024 manufacturing campaign for 5 faba bean concentrates, 1 pea protein concentrate and 1 faba bean isolate. • analytical compositions and masses of the pulse’s main and side streams (including wastes) • water (mass measurements), • energy (wattmeter or calculation), • cleaning products (technical data and mass) • materials and metals from time measurement and technical data. Foreground data were consolidated with literature [[1], [2], [3]] and expert say from Sylvie Dauguet (Terres Inovia). Compressed air production data from similar equipment at UMR STLO was used as a proxy. Background data were taken in the Ecoinvent 3.9 and Agribalyse 3.1.1 databases.
Data source location	Foreground data collected by Agri-Obtention cultivation farm • City/Town/Region: Orsonville • Country: France • Latitude: 48° 29′ 23.68″N and longitude: 1° 50′ 5.89″E
	Foreground data collected at the IMPROVE SAS pilot plant • City/Town/Region: Dury • Country: France • Latitude: 49° 52′ 25.21″N and longitude: 2° 16′ 20.50″E
	Foreground data stored by UMR 1253 Science et Technologie du Lait et de l’Œuf (STLO) • Institution: INRAE-Institut Agro • City/Town/Region: Rennes • Country: France • Latitude: 48° 6′ 55.33″N and longitude: 1° 42′ 29.01″W Background data from databases (selected proxies: France, if not: Europe and if not: Global)Agribalyse 3.1.1 • Institution: ADEME • City/Town/Region: Nantes • Country: France • https://agribalyse.ademe.fr Ecoinvent 3.10 • Institution: Ecoinvent • City/Town/Region: Zurich • Country: Switzerland • https://ecoinvent.org
Data accessibility	Repository name: DataInrae in https://entrepot.recherche.data.gouv.fr/ Data identification number: https://doi.org/10.57745/0G65L7 ref [4] Direct URL to data: https://entrepot.recherche.data.gouv.fr/privateurl.xhtml?token=e652564a-7c55–4b0b-b4b0–3c9b1581ae14 Instructions for accessing these data: please fill in the visitor guest-book
Related research article	None.

## 1 Value of the Data

• This dataset provides complete life cycle inventory (LCI) data for the production of plant-based protein concentrates or isolate on a semi-industrial scale, a realistic scale for 10- to 100-kg batches.
• LCI of food processing operations are comparatively less documented than LCI of farming activities due to industrial confidentiality at the food processing stage. The release of semi-industrial datasets, thanks to the activity of pilot-plant facilities, provides reasonable proxies for life cycle assessment (LCA) practitioners.
• LCI and LCA of plant-based products are comparatively less documented than those of animal-based products due to the major focus on the environmental impacts of breeding in the last 20 years [5,6]. The release of LCI and LCA data for the production of plant-based ingredients is important to allow a fair comparison between processed animal-based foods and their plant-based substitutes.
• LCA practitioners may use the data for the eco-design of processed food.

## 2 Background

Pulses have a crucial role to play in agroecological transitions due to their ability to transfer atmospheric nitrogen to nutritious proteins. However, they are underused in the French agri-food system due to agronomical and technological issues that require improvements from seed production to processing and consumption. In particular, pulses generate secondary metabolites that positively influence their resistance to biotic or abiotic stresses but are also associated with astringency, altered digestibility or even adverse nutritional effects of the food produced with them [[7], [8], [9]]. To provide the scientific and technical improvements to help develop this sector, the overall objective of the LETSPROSEED project is to reduce the occurrence of detrimental secondary metabolites in seeds by controlling genetic and agronomic factors (research axis 1) and/or to mitigate their adverse effects during food processing, consumption or digestion by controlling ingredient production and formulation into dairy product analogues (research axis 2 – see also https://letsproseed.hub.inrae.fr/ and https://anr.fr/ProjetIA-22-PLEG-0002).

LETSPROSEED includes a subtask for the quantitative assessment of the potential environmental impacts of the plant-based foods produced with the selected seeds (5 faba bean varieties and 1 pea variety) as part of the multiple dimensions of the overall quality of food. To do this, the potential environmental impacts of the pulse protein ingredients produced from the seeds is first needed. The objective of this dataset is therefore to release detailed LCI and LCA datasets of these ingredients for further evaluation of formulated foods, for the identification of environmental hotspots in the system and overall, for the eco-design of plant-based substitutes to animal-based products.

## 3 Data Description

The dataset contains all the data related to the LCIs and LCAs of semi-industrial production of 5 faba bean concentrates, 1 pea concentrate and 1 faba bean isolate. The term “semi-industrial” is used to mean that the unit operations used for manufacturing are the same as in the industry, while the scale of production was that of a pilot plant. The data are available in the following files:

1. Pulse_Protein_Concentrate_Production_System_Diagram: this is a figure file with 2 slides showing the system boundaries and the different unit operations considered for the semi-industrial manufacture of pulse protein concentrates (5 faba bean ones + 1 spring pea) at the IMPROVE SAS facility. The purpose of this file is to provide a comprehensive and detailed overview of the systems and to assign numbers to each unit operation so that it can then be linked to the tabulated data. Thus, the numbers in yellow boxes are those reported in files 2–6, 12 and 13.
2. LCI_Pea(C9861)_Process_diagram_and_Inventory_data_Concentrate: this is a table file with 10 sheets containing all the life cycle inventory data for the production of a pea protein concentrate from the C9861 (FURIOUS) variety. The first sheet presents a copy of the system (file 1) recalling the sequence and numbering of the 9 unit operations (including cleaning). The following 9 sheets present detailed and sourced LCI information related to each of the unit operation, for a reference flow of processing 72.8 kg of harvested seeds.
3. LCI_Faba(C9XXX)_Process_diagram_and_Inventory_data_Concentrate: these are 5 individual table files with 10 sheets each, similar to file 2. While file 2 is about the production of 1 pea protein concentrate, the present files are about the individual productions of 5 faba bean concentrates. The sheets and information are organized in the same way in each file. The code C9XXX corresponds to the variety (Table 1). The corresponding reference flows are as follows.

**Table 1:** Variety and reference flows of the 5 faba beans concentrates, coded as C9XXX.

Code	Variety	Reference flow
C9862	DIVA	Processing 91.8 kg of harvested faba bean seeds
C9863	NAIROBI	Processing 117.6 kg of harvested faba bean seeds
C9864	NAVARA	Processing 90.3 kg of harvested faba bean seeds
C9965	NAVI	Processing 103.6 kg of harvested faba bean seeds
C9896	VICTUS	Processing 87.6 kg of harvested faba bean seeds
4. LCI_CompressedAirProxy: this is a table file with 2 sheets containing the life cycle inventory (sheet 1) and life cycle impact assessment (sheet 2) for the production of 50 m^3^.h^-1^ compressed air by a STLO equipment, similar the one used at IMPROVE SAS (same brand, same range, different capacity). Input data were corrected using supplier technical data. The impact assessment was calculated using the Simapro Analyst software 9.5.0.1, the Ecoinvent 3.9 database and the EF 3.0 characterization method.
5. LCI_Processus_list_Concentrates: this is a table file with 3 sheets showing the explicit title of the Ecoinvent 3.9 or Agribalyse 3.1.1 database proxies used for the life cycle inventories of the pulse protein concentrate productions. The first sheet included all unit operations up to separating hulls and kernels, the second sheet included all unit operations up to separating the protein-rich light fraction from the starch-rich heavy fraction, and the third sheet is related to compressed air production. Each unit operation is called by its number in file 1.
6. LCI_Allocations_factors_Concentrates: this is a table file containing the calculated allocation factors at the air cleaning, dehulling, micronisation and classification steps of the protein concentrate production. Dehulling is presented with 2 options: the IMPROVE scenario where hulls were disposed of as a waste, and an industrially realistic scenario where hulls are valorized into feed or biomaterials. In the performed life cycle assessment, only the IMPROVE scenario was considered (i.e. no allocation on dehulling).
7. LCI_Calcul_Pedigree_All_Except_Spray-drying: this is a table file containing the Pedigree matrix and final self-evaluation of the data’s distribution, regarded as log-normal. This file is applicable to all unit operations for both concentrate and isolate productions, except for the spray-drying unit operation for which a separate evaluation was made due to lower quality of the foreground data.
8. Pulse_Protein_Isolate_Production_System_Diagram: this is a figure file with 4 slides showing the system boundaries and the different unit operations considered for the semi-industrial manufacture of one C9863 faba bean protein isolate at the IMPROVE SAS facility. The purpose of this file is to provide a comprehensive and detailed overview of the systems and to assign numbers to each unit operation so that it can then be linked to the tabulated data. Thus, the numbers in yellow boxes are those reported in files 9, 10 and 14.
9. LCI_Faba(C9863)_Process_diagram_and_Inventory_data_Isolate: this is a table file with 14 sheets, containing all the life cycle inventory data for the production of the C9863 faba bean protein isolate. The first sheet presents a copy of the system (file 8) recalling the sequence and numbering of the 12 unit operations (including cleaning). The following 13 sheets present detailed and sourced LCI information related to each of the unit operation, for a reference flow of processing 117.6 kg of harvested beans. Of note, operation 10 (stabilization) was split into 2 sheets for 10A (pasteurization) and 10B (spray drying).
10. LCI_Processus_list_Isolate: this is a table file with 4 sheets showing the explicit title of the Ecoinvent 3.8 or Agribalyse 3.1.1 processus or database proxies used for the life cycle inventories of the pulse protein isolate production. The first sheet included all unit operations up to separating hulls and kernels, the second sheet included all unit operations up to neutralizing and heating the globulin isolate slurry, the third sheet regarded spray drying only, the fourth sheet is related to cleaning of the wet line and the fifth sheet is related to compressed air production. Each unit operation is called by its number in file 8.
11. LCI_Calcul_Pedigree_Spray-drying: this is a 1-sheet table file containing the Pedigree matrix and final self-evaluation of the spray drying data’s distribution, regarded as log-normal one.
12. LCA_Pea(C6861)_Impacts_Contributions_Concentrate: this is a table file with 8 sheets showing the raw data of the potential environmental impacts for the production of 1 kg of pea protein concentrate, also known as pea protein “light fraction” (LF), calculated with the EF 3.0 characterization method in the SimaPro Analyst 9.5.0.1 software. The first sheet presents the calculated environmental impacts of 1 kg of the kernel intermediate product, issued from step 1 (upstream agricultural production and delivery) to 4 (dehulling). Following the IMPROVE scenario, no allocation was applied on dehulling. The first sheet also presents the calculated environmental impacts for the mass of the intermediate product that is precisely required to eventually manufacture 1 kg of the final pea protein concentrate, based on the contribution of the kernel ingredient to the overall environmental impacts of 1 kg concentrate as calculated for each allocation factor (sheet 2). Since allocation factors were similar or even equal for the dry-matter or the mass allocations, only one LCA table is reported. The third, fourth and fifth sheets present the full detail of all individual contributions of all unit operations from step 1 to step 9 (i.e. cradle to gate), by dividing the contribution of the kernel intermediate product in sheet 2 into its own individual unit operations (from sheet 1) when respectively applying dry matter/mass, protein or economic allocation at the classification step. The sixth sheet presents an aggregated form of these detailed LCA data, to better evidence the contributions of compressed air, electricity, machines, transportation, packaging, tap water and waste management at each step. The seventh sheet shows comparison with database proxies. The last sheet presents the uncertainties of the calculated environmental impacts, using 1000-run Monte-Carlo simulation that provided statistical data with 95 % confidence: mean, median, standard deviation, coefficient of variation, and the standard error of the mean.
13. LCA_Faba(C9XXX)_Impacts_Contributions_Concentrate: these are 5 individual table files with 8 sheets each, shaped similarly as file 12. The code C9XXX corresponds to the variety.
14. LCA_Faba(C9863)_Impacts_Contributions_Isolate: this is a table file with 12 sheets showing the raw data of the potential environmental impacts for the production of 1 kg of C9863 faba bean protein isolate, calculated with the EF 3.0 characterization method in the SimaPro Analyst 9.5.0.1 software. As for files 12 and 13, the first four sheets present the calculated environmental impacts of 1 kg of the intermediate products (kernels and globulin slurry) and of the final product including cleaning. Contributions of the intermediate products were also calculated for their precise contributions into the final product, as to present the full detail of all individual contributions of all unit operations from step 1 to step 12 except the final delivery 11 (i.e. cradle to gate). These data were presented with 2 (low or high) levels of input data for the energy consumption of spray drying. As for files 12 and 13, the next sheets show the aggregated form of these detailed LCA data, to better evidence the contributions of compressed air, electricity, machines, transportation, packaging, tap water and waste management at each step. The last sheets present the uncertainties of the calculated environmental impacts, using 1000-run Monte-Carlo simulation that provided statistical data with 95 % confidence: mean, median, standard deviation, coefficient of variation, and the standard error of the mean. The sheet “C9863 ingredients” compares the environmental impacts of the isolate with those of the concentrate, as calculated in file 13 using dry matter, mass, protein or economic allocation.
15. LCA_All_seeds_(DM): this is a table file with 3 sheets showing the contributions of agricultural production and of transformation (processing) in the environmental impacts of 1 kg of each of the pea or faba bean protein concentrates.

## 4 Experimental Design, Materials and Methods

This work followed the International Standard Organization (ISO) recommendations for applying the life cycle assessment (LCA) method [10].

### 4.1 Goal and scope

The goal of this study is to assess the environmental impacts of the production of 5 faba bean protein concentrates, 1 pea protein concentrate and 1 faba bean protein isolate at semi-industrial scale. The scope is that of typical processes for the preparation of pulse-based food ingredients in the French industry in the 2020s [11,12].

### 4.2 System boundaries

The system boundaries are cradle to gate, including agricultural production at the farm, post-harvest treatment of the seeds, transport to the IMPROVE pilot plant, processing and packing at the IMPROVE pilot plant and delivery of the product to Dijon where it was meant to be used. Typically, pulse-based protein concentrates are made from harvesting of raw mature seeds, followed by cleaning, sorting, dehulling, grinding, micronisation, air classification and packaging (so-called “dry” processing route). Meanwhile, pulse-based protein isolates are typically made from the flour resulting from grinding, which is dispersed in water and subjected to isoelectric precipitation, separation and washing cycles in order to isolate and dry a soluble globulin-rich fraction (so-called “wet” processing route). Cleaning of the processing line is included. Except from the starch-rich coproduct of pulse protein concentrates, all side streams were regarded as wastes. Downstream supply chain, use and consumption of the product are excluded. Regarding the IMPROVE facility, the overall building and associated resources for labor (heating, catering, transport…) as well as support activities (maintenance, quality, R&D…) are excluded.

### 4.3 Functional unit

The functional unit was 1 kg of pulse-based ingredient powder produced at a semi-industrial scale and packed in 5-kg kraft paper bags at the IMPROVE pilot plant facility.

### 4.4 General description of the system

**Fig. 1:**
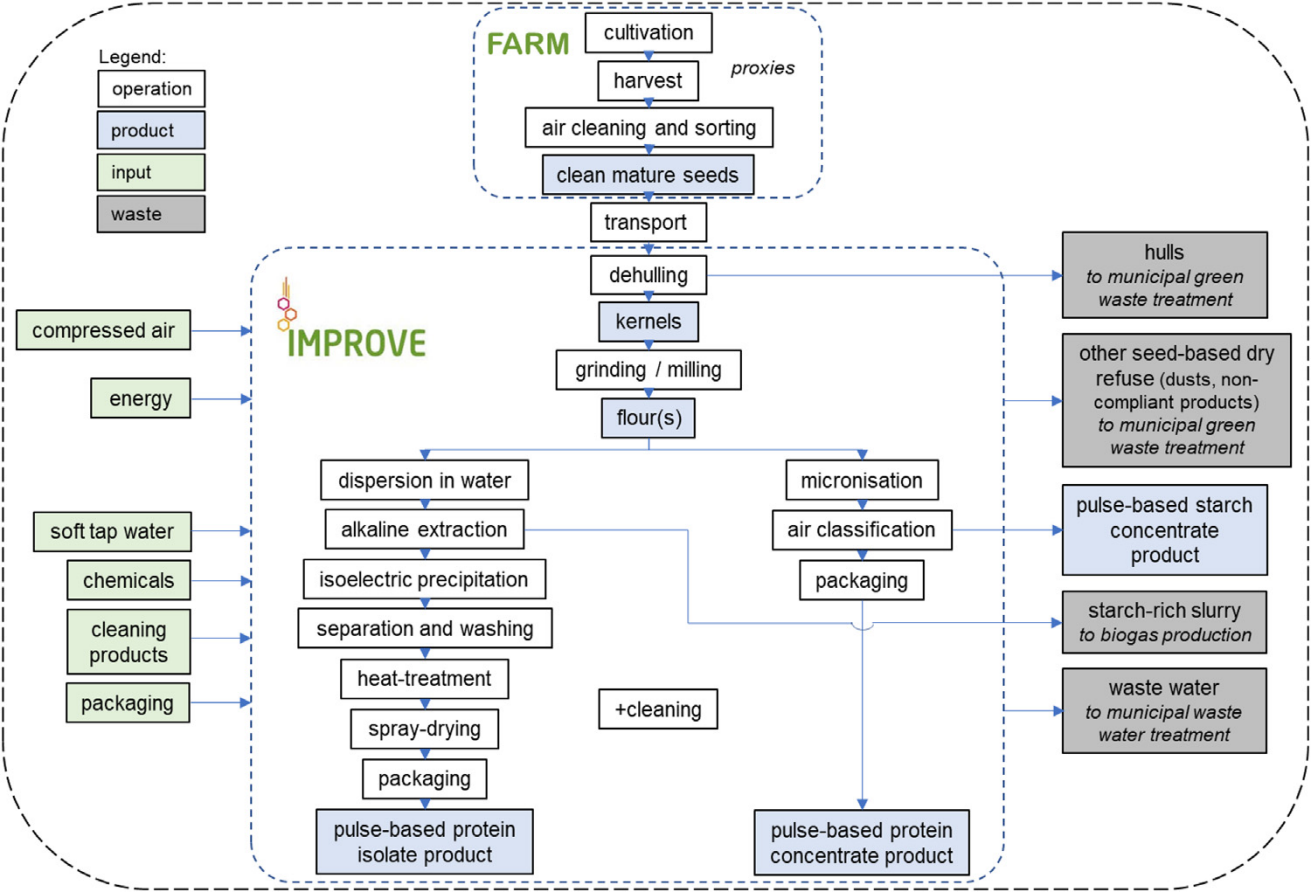
System of the pulse-based food protein ingredient semi-industrial production under study. End of life scenarios are indicated in italics for each waste output stream. Hulls, other seed-based dry refuse and waste water can exit either the dry (concentrate) or wet (isolate) processing lines. Differences with the industrial scale are discussed in the limitations sectio*n.*

Four faba bean and one pea varieties were from the plant breeder Agri-Obtention (Guyancourt, France) and only one variety (faba bean C9896 VICTUS) was from RAGT (Rodez, France). These varieties were cultivated using conventional farming practices and the Orsonville Agri-Obtention farm (France) was taken as a location proxy. Once harvested, the seeds were air cleaned and sorted using a JCC 05 JK Machinery (Praha, Czech republic – IMPROVE proxy). They were then transported by truck to IMPROVE (Dury, France), where they were processed into 6 protein concentrates (all varieties) and 1 protein isolate (faba bean C9863 – Fig. 1).

Dehulling was operated by a sequence using an Alma Pro 100 stone mill (Saint Donnat sur Herbasse, France), a Quasar 1000 optical sorter (Argelato, Italy) and the JCC 05 air sorter. Grinding was done using a Retsch SM 300 knife mill (Haan, Germany), while micronisation and air classification were performed on a Hosakawa Alpine combination of a 70 ZPS impact mill and a 70 APS classifier, respectively set at 3800 rpm for micronisation and 9000 rpm for classification (Hosokawa Alpine Group, Augsburg, Germany). The resulting protein concentrate was then packed in 5-kg kraft bags. For the production of the C9863 faba bean isolate, the ground kernels were milled using a Bühler MLU 202 roller mill (Uzwill, Switzerland). The resulting flour was dispersed at a flour/ratio of 1/6 w/w into soft tap water at room temperature, in a stainless-steel tank, agitated and alkalinized using NaOH 1 M. Insoluble material (fibers and starch) were eliminated using a Flottweg Z23 decanter (Vilsbiburg, Germany) then an Easyscale GEA clarificator (Düsseldorf, Germany). The clear supernatant, containing the albumin and globulin proteins, was then acidified using HCl 1 M to precipitate the globulin protein at their isoelectric point (pH 4–5) in an agitated stainless-steel tank. The precipitate is recovered using the same GEA separator, washed with acidified tap water then separated again, neutralized using 1 M NaOH, pasteurized at 75 °C for 15 s with a HT 200 tubular heat exchanger (OMVE, De Meern, The Netherlands) and finally spray dried without preliminary vacuum concentration on a simple effect SiccaDania spray dryier (Birkerød, Denmark). The spray drying parameters were unknown but similar operations, measured at UMR STLO or taken in the literature, were used to assume whether a low-energy (proxies) or high-energy (equipment’s nominal power) consumption levels. The resulting protein isolate was then packed in 5-kg kraft bags.

For the 1-day production of the pulse-based protein concentrates, cleaning involved compressed-air dust removal then final rinsing of the line with soft tap water. For the 2-day production of the C9863 protein isolate, cleaning involved dust removal using compressed air for all “dry” unit operations as for the concentrate production stream, as well as cleaning in place (CIP) of all “wet” unit operations using 3 % NaOH 1 M at 80 °C. Considering the lifetime of the alkaline stock solution, only a 2-day share of the weekly discharge and replacement was input to the product. The wet unit operations were rinsed with soft tap water prior to and after CIP cleaning.

## 5 Life Cycle Inventory

### 5.1 Seeds

Database proxies for French conventional agricultural productions of spring faba bean or protein pea seeds were taken from Agribalyse 3.1.1 or Ecoinvent 3.9, respectively. The average yield of 5 tons.ha^-1^ given for the faba bean proxy was corrected to 3 tons.ha^-1^ for the faba bean varieties C9862 and C9965 as they performed less than the three other varieties.

### 5.2 Masses and compositions

In general, all input and output flows were weighted to ensure primary data for mass balance verification. Only gases were either omitted (nitrogen during air classification) or calculated as time and flow-rate (compressed air). Dry matter of the pulse-based inputs, intermediate and final products was measured by IMPROVE using an internal standard method. Briefly, the principle is to precisely weight a sample and to dry it in an oven at ∼105 °C until weight at room temperature is constant. The total protein content was determined by IMPROVE using the standard Kjeldhal nitrogen determination method and a nitrogen-to-protein conversion factor of 6.25. As contents were only used for mass balance, this factor was not challenged [13].

### 5.3 Water

The Ecoinvent proxy for “tap water, Europe without Switzerland, market for” was taken for all water inputs of the system. Softening was neglected. All output wet side streams were regarded as wastewater, except for the starch-rich slurry from alkaline extraction in the wet processing of faba bean isolate, which was directed to biogas production (see section 5.10).

### 5.4 Compressed air

Compressed air at IMPROVE was produced by a Rollair 1400 single compressor (Worthington Creyssensac, Cergy-Pontoise, France) nominally serving ∼85 m^3^.h^-1^. As primary data was difficult to collect on-site, the UMR STLO Rollair 1000 dual compressor was taken as a proxy, for a nominal service of ∼50 m^3^.h^-1^ compressed air at 8 × 10^5^ Pa. Consumption was calculated *prorata temporis* in the life cycle inventory of the present pulse-based ingredients. This consumption was most likely under-estimated, as the capacity of the STLO equipment was less. The Ecoinvent proxy for “Compressed air, 800 kPa gauge {RER}| market for compressed air, 800 kPa gauge | Cut-off, U” was also tried for comparison and yielded 4 to 10-fold greater impacts than the primary Rollair 1000 inventory data. Furthermore, the compressors work on demand and it was not possible to measure when the reservoir needed refill and how much for and/or how much compressed air is really consumed on processing the pulse-based ingredients. Time-based use of the Rollair 1000 was therefore considered a best compromise.

### 5.5 Equipment

The masses of the different materials that composed each equipment were taken in manufacturers’ technical data. The structures were essentially in stainless chromium steel. Proxies for global 18/8 chromium steel production and metal working product manufacturing were used as listed in the dataset. Wearing parts (knifes, wooden frames, rings…) were neglected except for the mill’s stone. The reference flow for each equipment was calculated *prorata temporis* of a 30-year lifetime.

### 5.6 Packaging

The cleaned seeds were transported from Agri-Obtention to IMPROVE in 25-kg kraft paper bags with a low-density polyethylene internal sleeve. On processing, the intermediate dry products such as kernels or flour were stored from one to the next operation unit in high-density polyethylene drums with steel circle closure. Wet intermediate products were kept in stainless steel tanks (regarded as equipment). The final powders were packed in 5-kg kraft paper bags.

### 5.7 Energy

IMPROVE’s equipment was powered with electricity from the grid (French mix). Electrical consumption of three-phase engines and pumps (cosφ ∼0.8) was calculated using the following Eq. (1), knowing either the *P* (kW) or *S* (*V* × *A*) nominal power and time use *t* (h):

(1) $$E\left(kWh\right)=P\left(kW\right)\times t\left(h\right)=U\left(V\right)\times I\left(A\right)\times \sqrt{3}\times \text{cos}\varphi \times t\left(h\right)\times 10^{-3}$$

Heating water for cleaning or pasteurization used natural gas. Energy consumption for heat transfer to a mass *m* (kg) of water or of a dilute protein solution was calculated as (Eq. (2)):

(2) $$E\left(\text{kWh}\right)=m\times \text{Cp}\times \Delta \theta \times {\left(3.6\times \;10^{6}\right)}^{-1}$$

where Cp is the heat capacity of water (4180 J.K^-1^.kg^-1^) and Δθ the difference between the target and the starting temperatures of the product (in K).

### 5.8 Transport

Transport of the seeds from field to farm was assumed to be done with a tractor and two-axle trailer. Transport of the cleaned seeds from Agri-Obtention to IMPROVE was assumed to be done in a EURO VI 3.5–7.5 m^3^ lorry at ambient temperature.

### 5.9 Prices

Prices of the pulses or waste flows were given by experts at IMPROVE.

### 5.10 End of life scenarios

All solid pulse-based waste streams (i.e. hulls, dusts…) were treated as municipal green waste. The starch-rich slurry from the faba bean protein isolate production was sent to biogas production, while all other wet side streams or cleaning effluents were sent to municipal wastewater treatment.

### 5.11 Life cycle impact assessment

The life cycle assessment of the pulse-based ingredients’ potential environmental impacts was performed using the Environmental Footprint 3.0 characterization method (EF 3.0 adapted version 1.03) using the SimaPro Analyst software release 9.5.0.1 (PRé Sustainability, Amersfoort, The Netherlands) and the Agribalyse 3.1.1 [14] and EcoInvent 3.9 [15] databases. These tools were all provided by the INRAE-CIRAD Multi-Criteria Assessment of Sustainability platform (MEANS).

## Limitations

The LETSPROSEED research project involves screening of faba bean varieties for nutritional and techno-functional performances. A national average proxy was taken for faba bean or pea production, only roughly corrected for the two faba varieties that exhibited low yield. Therefore, the present life cycle inventory misses primary data for agricultural production, which exhibits the greatest contribution to the ingredient’s environmental impacts, as usually expected for food products [5]. On the other hand, the dataset provides a good sensibility analysis of the environmental impacts of processing, through the natural variety of the 5 faba bean varieties.

IMPROVE SAS is a semi-industrial facility and therefore lacks some of the economies of scale that could be typically found in the industry. Daytime operation hours (instead of 3 × 8 h) were compensated by considering depreciation only for the duration of actual manufacturing of the ingredients. However, an industrial facility would not waste hulls and allocation factors are provided for those whom would want to consider hulls as a coproduct. Energy and water consumptions for ramping up or cleaning operations are also input on smaller batches of product than in the industry, as discussed in Gaillard et al. [3]. An industrial spray drying equipment would include a pre-concentration unit and possibly a second effect to cut down energy costs. Finally, some end of life scenarios cannot be put in place when the output stream is small, e.g., directing pulse-based dusts and refuses to feed.

Finally, some limitations exist in the primary data collected. Maintenance costs were omitted. They are 2.5 % of the total running costs of equipment and include consumables, repair, energy and labor. Electricity and heat consumptions were only deduced from calculation and not measured. It was hypothesized that engines worked at their nominal power and that heat losses were negligible both on pasteurization or on storage of the hot cleaning solution. The quality of the data for electrical consumption of the spray drier was relatively poor, as calculation also implies uncertainty on the actual consumption for pre-heating and drying the input air. This was considered in the Pedigree matrix as well as in the life cycle inventory of the isolate production.

## Ethics Statement

The authors state that the current work did not involve human subjects, animal experiments, or any data collected from social media platforms.

## CRediT Author Statement

**Fanny Guyomarc’h:** conceptualization, methodology, investigation and resources, data curation, writing – original draft, writing – review and editing; **Simone Scussat:** conceptualization, investigation and resources, writing – review and editing; **Jean-Eudes Hermant:** conceptualization, investigation and resources, writing – review and editing; **Claire Berton-Carabin:** conceptualization, supervision, writing – review and editing; **Marc Anton:** conceptualization, supervision, writing – review and editing; **Karine Gallardo-Guerrero:** conceptualization, supervision, funding acquisition, writing – review and editing; **Rémi Saurel:** conceptualization, investigation and resources, supervision, writing – review and editing.

## Acknowledgements

This research was funded by France 2030 as part of the ANR-22-PLEG-0002 “LETSPROSEED” project selected by the National Research Agency (ANR, Paris, France). The authors thank Agri-Obtention and RAGT for supplying the seeds. Fanny Guyomarc’h (UMR STLO) thanks Sylvie Dauguet (Terres Inovia, Pessac, France) for literature recommendations on life cycle environmental impact assessment of plant-based ingredients.

## Data Availability

DataGouvTask 3.4 - Deliverable 1 - Life cycle assessment of the LETSPROSEED pulse protein ingredients (Original data).
